# Sepsis in silico: definition, development and application of an electronic phenotype for sepsis

**DOI:** 10.1099/jmm.0.001986

**Published:** 2025-03-28

**Authors:** Zahraa Al-Sultani, Timothy JJ Inglis, Benjamin McFadden, Elizabeth Thomas, Mark Reynolds

**Affiliations:** 1 School of Physics, Maths and Computing, Computer Science and Software Engineering, University of Western Australia, Crawley, WA 6009, Australia; 2 Division of Pathology and Laboratory Medicine, School of Medicine, University of Western Australia, Crawley, WA 6009, Australia; 3 PathWest Laboratory Medicine WA, QEII Medical Centre, Nedlands, WA 6009, Australia; 4 Curtin School of Population Health, Curtin University, Bentley, WA 6845, Australia

**Keywords:** bloodstream infection, digital phenotype, machine learning, precision medicine, sepsis

## Abstract

Repurposing electronic health record (EHR) or electronic medical record (EMR) data holds significant promise for evidence-based epidemic intelligence and research. Key challenges include sepsis recognition by physicians and issues with EHR and EMR data. Recent advances in data-driven techniques, alongside initiatives like the Surviving Sepsis Campaign and the Severe Sepsis and Septic Shock Management Bundle (SEP-1), have improved sepsis definition, early detection, subtype characterization, prognostication and personalized treatment. This includes identifying potential biomarkers or digital signatures to enhance diagnosis, guide therapy and optimize clinical management. Machine learning applications play a crucial role in identifying biomarkers and digital signatures associated with sepsis and its sub-phenotypes. Additionally, electronic phenotyping, leveraging EHR and EMR data, has emerged as a valuable tool for evidence-based sepsis identification and management. This review examines methods for identifying sepsis cohorts, focusing on two main approaches: utilizing health administrative data with standardized diagnostic coding via the International Classification of Diseases and integrating clinical data. This overview provides a comprehensive analysis of current cohort identification and electronic phenotyping strategies for sepsis, highlighting their potential applications and challenges. The accuracy of an electronic phenotype or signature is pivotal for precision medicine, enabling a shift from subjective clinical descriptions to data-driven insights.

## Introduction

Sepsis is a complex syndrome characterized by physiological, biological and biochemical abnormalities resulting from an imbalanced host response to infection. This can lead to multiple organ dysfunction syndrome and death [1,2] and septic shock, a life-threatening condition marked by a critical drop in blood pressure. Currently, sepsis diagnosis relies on a combination of clinical signs and symptoms, as no single definitive test exists. Efforts have been made to establish clear, valid clinical criteria for sepsis diagnosis. Timely medical intervention is crucial to address the diverse clinical presentations and prevent progression to more critical stages. The definitions of sepsis and septic shock have significantly evolved since the early 1990s [1,3,7].

The International Classification of Diseases (ICD), overseen by the World Health Organization (WHO), serves as a global system for coding medical diagnoses, symptoms, procedures and other health-related information in administrative data. The ICD is periodically reviewed to reflect changes in clinical and research settings. Currently, ICD-10 is widely used, standardizing the recording, analysis and comparison of health data worldwide [8]. Countries often modify the base version of ICD-10 to their specific needs, such as the Australian Modification (ICD-10-AM), maintained by the Independent Health and Aged Care Pricing Authority [9], and ICD-10-CM in the USA, managed by the Centers for Medicare and Medicaid Services (CMS) and the National Center for Health Statistics [10]. Despite the introduction of ICD-11 on 1 January 2022, the WHO acknowledges a gradual global transition, recognizing that many countries lack the capacity to adapt quickly to the new classification system [8].

Analysing hospital administrative data is one method of understanding the burden of sepsis within a given jurisdiction or region [11]. These data include precise records of healthcare usage, procedures and costs, gathered primarily for administrative purposes but also utilized to study healthcare delivery and costs [12]. However, these data are limited by differing and evolving coding practices, policies, financial incentives and documentation methods. An alternative approach to assessing sepsis incidence and trends is the use of objective health data available in electronic health records (EHRs). These records include blood cultures, prolonged antibiotic use for infection identification and indicators, such as laboratory values, vasopressor use and mechanical ventilation, to assess acute organ dysfunction [11]. Machine learning (ML) in sepsis research involves supervised learning with labelled data, semi-supervised learning to reduce the need for labelled data, weakly supervised learning to minimize reliance on gold-standard labels and unsupervised learning for unveiling hidden patterns in high-dimensional datasets. These methodologies collectively contribute to a comprehensive exploration of sepsis, with supervised learning crucial for many applications and unsupervised learning excelling in defining distinct phenotypes.

This review explores sepsis cohort identification and electronic phenotyping, highlighting challenges in defining sepsis and managing dynamic, incomplete and disparate EHR data. It underscores ML’s dual role in predicting sepsis and refining data labelling and dimensionality reduction for model training. Moreover, it plays a pivotal role in identifying distinct sepsis subtypes, offering insights for tailored and effective treatment responses.

## What is sepsis?

The most recent definition of sepsis, known as Sepsis-3, was announced at the 45th Critical Care Congress at the beginning of 2016 and defines sepsis as ‘life-threatening organ dysfunction caused by a dysregulated host response to infection’. Sepsis is a serious condition that can lead to septic shock, a subset of sepsis in which circulatory and cellular/metabolic abnormalities are profound enough to substantially increase mortality [1].

Infections caused by bacteria, viruses, parasites and fungi can lead to sepsis when the body cannot control the infection, resulting in damage to its own tissues and organs [13,14]. While bacterial infections are the main cause, fungal or viral infections can also trigger sepsis [14].

**Fig. 1.**
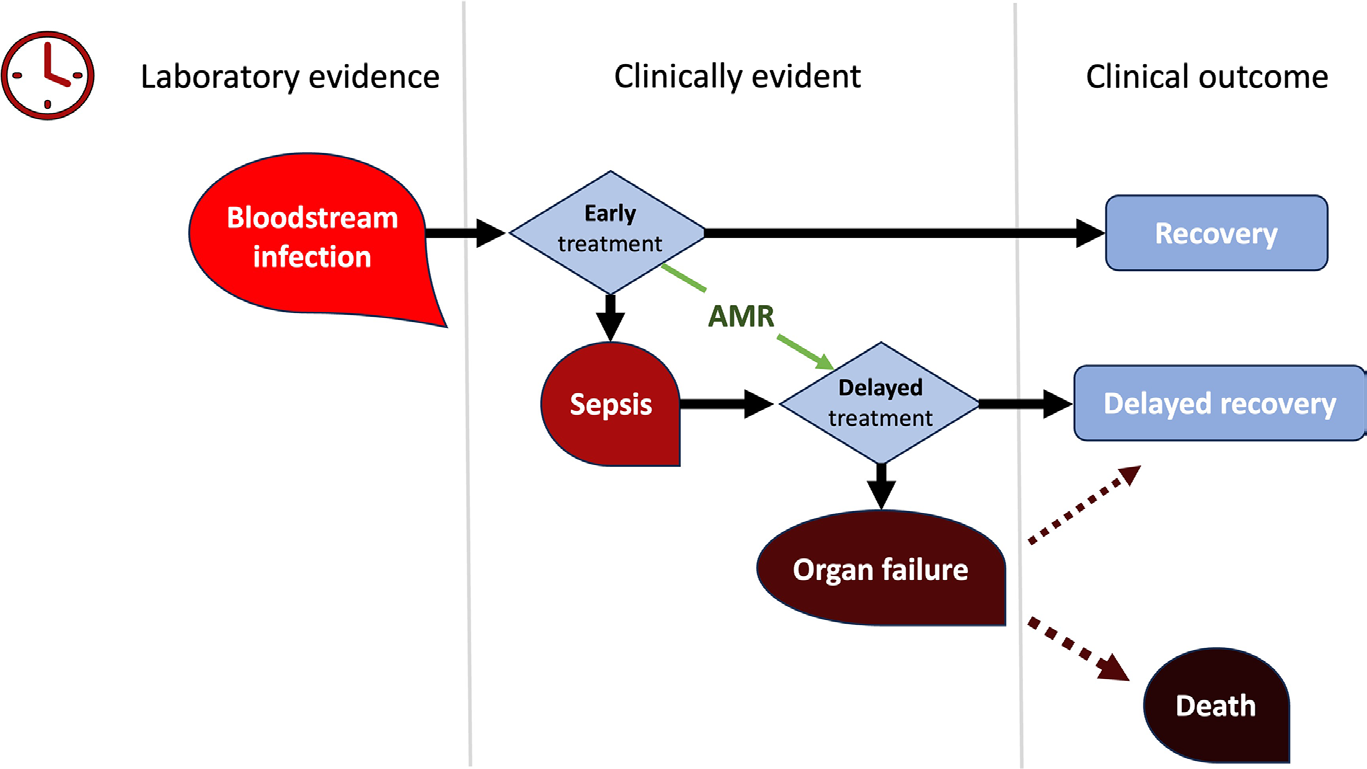
Schematic diagram illustrating BSIs, sepsis and their time-sensitive nature, exacerbated by antimicrobial resistance, delaying optimal therapy, adapted from Inglis [16].

The onset of sepsis can be traced back to an infection anywhere in the body. Patients managed in the community or those already receiving medical care in a hospital might develop sepsis, whether post-surgery or arising from a localized infection. Sepsis can result from infections that spread throughout the body via the bloodstream [15]. Although sepsis and bloodstream infections (BSIs) share similarities, they are distinct clinical entities. BSIs can be confirmed through microbiological cultures, but their progression to sepsis is not guaranteed, as shown in Fig. 1. Conversely, sepsis can occur without a positive blood culture due to various factors [16].

Pre-existing conditions, such as chronic obstructive pulmonary disease, cancer and cirrhosis, AIDS and other immunodeficiency disorders increase the risk of sepsis [17]. Sociodemographic factors, including socioeconomic status, gender, race and lifestyle choices such as tobacco and alcohol use, also influence sepsis prevalence [17,19]. Access to high-quality healthcare, particularly intensive care, correlates with lower sepsis incidence and mortality rates [17].

Early diagnosis and treatment prevent severe outcomes such as septic shock, multiple organ failure and death. However, diagnosing sepsis can be challenging, especially in remote areas with limited access to diagnostic tools such as laboratory tests. Accurate, efficient and cost-effective methods for early sepsis detection are needed, particularly in high-risk populations in remote areas.

The sepsis paradigm is evolving beyond emergency departments and intensive care units (ICUs) to encompass broader public health concerns. Population-based solutions are needed to address this shift. Future efforts in primary, secondary and tertiary prevention can benefit from identifying modifiable risk factors in high-risk groups [20].

Recent advancements in data-driven techniques have significantly improved the definition, early identification, sub-type characterization and management customization for sepsis patients.

## Burden of sepsis

In 2017, the WHO acknowledged sepsis as a global health priority [21]. Estimating the global impact of sepsis is technically demanding. A recent study by Rudd *et al.* [22] projected ∼48.9 million cases and 11 million sepsis-related deaths in 2017, representing ~20% of all global deaths. In U.S. hospitals, up to half of all hospital deaths are caused or exacerbated by sepsis [23,24].

In Australia, sepsis is a leading cause of morbidity and mortality. There are 55,000 cases and at least 8,700 deaths annually. The burden of disease is notably high for people living in very remote areas with limited healthcare access, particularly those facing socioeconomic disadvantage. Population groups at higher sepsis risk include the elderly, infants and individuals with compromised immune systems or chronic medical conditions. In 2021, the direct hospital cost of sepsis in Australia was $700 million, with an additional $4 billion in indirect costs [25,26].

Sepsis survivors often experience functional limitations hindering their return to work. A study by Skei *et al.* [27] explored return-to-work (RTW) outcomes for sepsis-hospitalized patients using data from the Norwegian Patient Registry linked to sick-leave records from the Norwegian National Social Security System Registry. The study found that returning to work remains challenging for many patients even 2 years post-discharge. More patients returned after 1 year compared with 6 months and 2 years. Factors associated with sustained RTW in sepsis survivors include younger age, fewer existing health conditions and less severe organ dysfunction during the acute phase.

## Challenges around sepsis: definition and diagnosis

Sepsis is a complex and dynamic syndrome with diverse presentations that evolve over time. Its diagnosis is affected by multiple definitions and evolving evidence, and the lack of a single definitive test. Consequently, a variety of screening tools and biomarkers are required [28].

The prevailing concept of sepsis (Sepsis-1) was established more than two decades ago, encompassing both systemic inflammatory response syndrome (SIRS) and the presence of suspected or confirmed infection [7]. Sepsis-2, introduced in 2001, recognized the diversity within the disease process and categorized sepsis into three stages: sepsis, severe sepsis and septic shock. In this context (framework), severe sepsis was described as sepsis accompanied by organ dysfunction, while septic shock featured persistent hypotension without an obvious alternative cause. However, the Sepsis-2 criteria can be challenging to recall under pressure, making them less ideal for use in busy clinical settings [29]. Sepsis-3, introduced in 2016, is the latest definition and relies on sequential organ failure assessment (SOFA) scores in conjunction with infection criteria. Sepsis-3 regards sepsis as a life-threatening condition more broadly, eliminating the category of severe sepsis [1]. Nevertheless, there has been criticism of the Sepsis-3 criteria due to the increased risk of identifying sepsis at a later stage in its clinical progression [30].

Since 1991, when sepsis gained wider global attention, four Surviving Sepsis Campaign recommendations have been issued. The international SSC, a collaboration between the Society of Critical Care Medicine (SCCM) and the European Society of Intensive Care Medicine (ESICM), has committed to improving the recognition and treatment of sepsis and septic shock as major causes of worldwide mortality. Additionally, SCCM aims to improve outcomes for sepsis survivors, including those affected by post-sepsis syndrome. Initiated through the Barcelona Declaration at the ESICM’s annual meeting in 2002, the campaign expanded through various phases, including the publication of four editions of evidence-based guidelines, the implementation of a performance improvement programme and the analysis of data from over 30,000 patient records collected worldwide [31].

In 2015, the CMS introduced the SEP-1 bundle to promote high-quality, cost-effective care through a comprehensive performance measure for sepsis. This protocol combines various treatments delivered over 3 and 6 h intervals for patients diagnosed with severe sepsis or septic shock [32]. In 2018, CMS released an updated version of its sepsis guidelines, also called SEP-1, maintaining the bundle approach. It is worth noting that CMS continues to use the Sepsis-2 definition, which encompasses sepsis, severe sepsis and septic shock, adding to the potential confusion [33]. The sepsis bundles provided by the CMS offer guidelines for clinicians, allowing the flexibility to implement or not based on experience and judgement. In contrast, the SEP-1 mandate is tied to hospital reimbursement, compelling clinicians to adhere to specific protocols [32].

## Data sources and electronic phenotyping

Electronic medical records (EMRs) and electronic health records (EHRs) are sometimes used interchangeably, yet they differ in scope. EMRs contain clinical data from specific providers or clinics, whereas EHRs provide a comprehensive view of a patient’s overall health from multiple providers. EHRs are designed to be shared among healthcare providers, unlike EMRs. Both EHR and EMR data comprise a mixture of unstructured and structured components. EHRs encompass various categories of individual patient-specific information obtained during clinical care and include, but are not limited to, any combination of demographics, diagnostic and procedure codes, vital signs, laboratory results, imaging, medications and physician notes. These longitudinal data are valuable for understanding disease development, progression and treatment responses over repeated clinic visits [34].

The availability of EHR datasets has enabled environmental and social epidemiologists to utilize data from patients situated in diverse physical, built and social environments. As patient addresses undergo regular checks and updates for billing and communication, researchers can easily link geocoded addresses to location-specific data and employ Geographic Information Systems to investigate an individual’s proximity to disease-related hazards. This approach allows for the examination of adverse health effects stemming from direct exposures like air pollution, as well as contextual exposures such as poverty rates within residential zip codes [35].

Identifying patients with specific conditions or outcomes, a process known as phenotyping, poses a fundamental challenge in using newly available EHR data. EHR-based phenotyping methods, crucial for efficient and accurate phenotype identification in EHRs, can be categorized into two types. Validated algorithms, designed for specific phenotypes using various EHR data sources, represent one category. In contrast, high-throughput methods aim to define a wide range of phenotypes through automation. Phecodes belong to the latter category. They involve the manual curation of ICD codes to capture meaningful concepts for research [36].

The development of phenotype algorithms is a highly iterative process that involves collaboration among researchers, clinicians, clinical support staff and information technology teams. This collaborative approach aims to gain a comprehensive understanding of the context in which data were captured at the point of care, allowing valuable insights to inform the development of algorithms. Input from each stakeholder group helps discern the nuances and intricacies of data collection, ensuring that the algorithms accurately capture the intended phenotypic characteristics. By incorporating diverse perspectives and expertise, the iterative nature of the process ensures the continuous refinement and enhancement of phenotype algorithms, ultimately leading to more precise and reliable results [37].

**Fig. 2.**
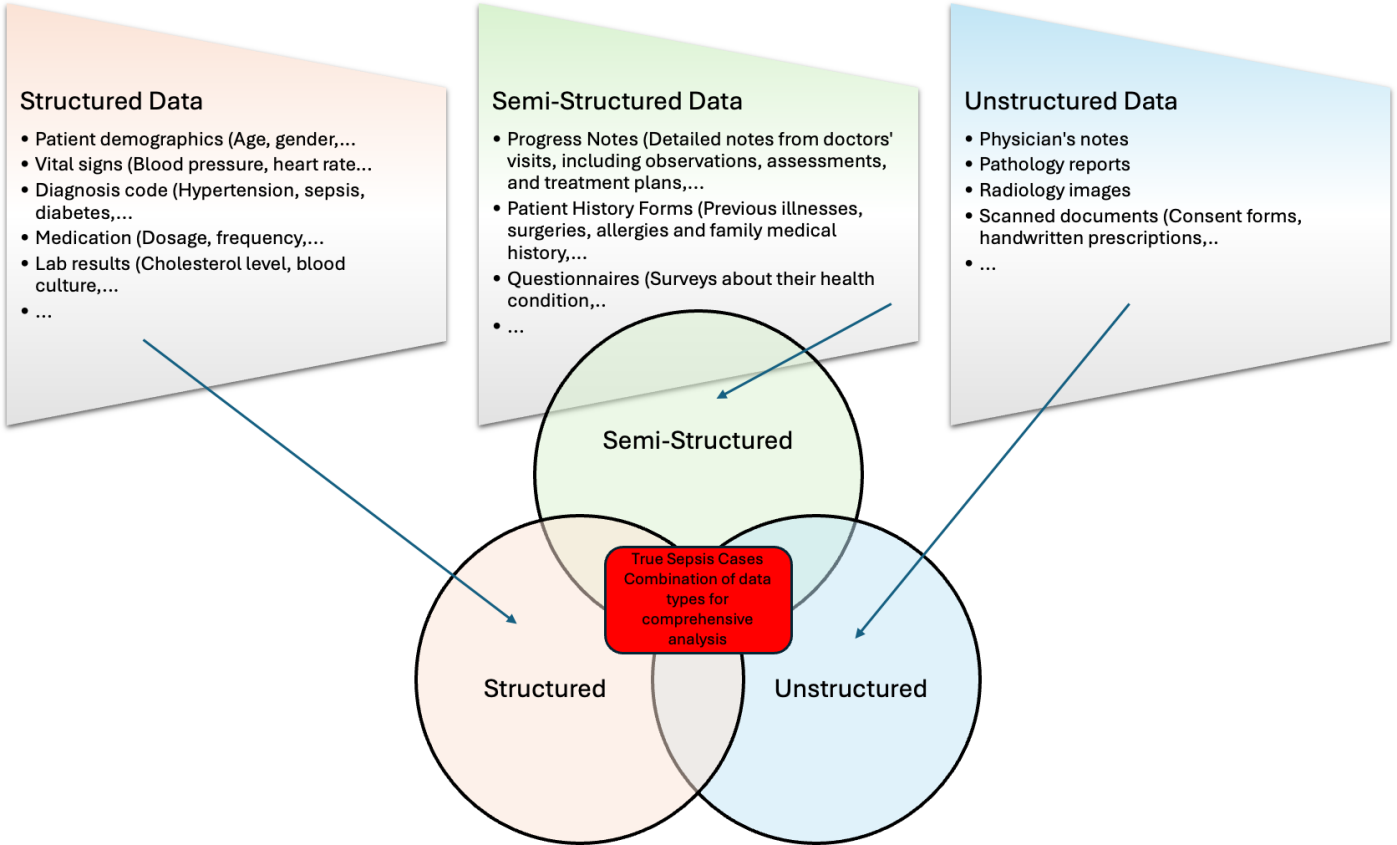
EHR data structure and phenotyping adapted from Wei and Denny [38].

Accurate phenotyping requires extracting information from various sources, including vital signs, laboratory results, diagnosis codes, progress notes and physician’s notes, regardless of their structure [38]. Integrating structured and unstructured data can significantly enhance the accuracy of ICD-only approaches, improving the validity of retrospective surveillance and cohort studies, and facilitating efficient real-time case identification in both clinical research and practice [39]. Fig. 2 shows that EHR data can be structured, semi-structured or unstructured, with structured data being easily retrievable and unstructured data requiring a more diverse range of tools [38].

## Challenges around electronic phenotyping: data and sepsis

Identification of phenotypes within EHRs is a complex informatics challenge due to the heterogeneous, incomplete and dynamic nature of the data. EHRs contain structured information, such as demographics, diagnosis codes, procedure codes, laboratory values and medication exposures, alongside unstructured data such as progress notes, discharge summaries and imaging/pathology reports. Data are irregularly distributed over time and fragmented across multiple institutions, with variations in data input methods and accuracy. Moreover, EHRs operate as dynamic systems influenced by clinical workflows, where the current state impacts future states. As a result, electronic phenotyping requires sophisticated methods that can handle record heterogeneity, integrate various data types and capture the intricate knowledge representation embedded within EHRs. The development of portable phenotypes usable across different sites poses an additional challenge [40].

On the other hand, sepsis, as a clinical diagnosis, presents a challenge due to the rapid changes in biomarkers. The goal is to provide evidence-based care for this highly complex condition [41]. A rule-based phenotype for sepsis typically involves defining criteria based on clinical indications and diagnostic codes. However, it is important to note that rule-based definitions have limits, as they cannot fully capture the complexity of sepsis and may require periodic updates as new evidence emerges [42].

## Obtaining labelled data in the medical field

Obtaining labelled data, which involves categorizing and annotating information, in the medical field is challenging, expensive and time-consuming. Currently, ML algorithms are primarily trained on data labelled using claims-based methods like ICD coding [43]. However, these methods have significant limitations. Labels derived from claims-based approaches exhibit high specificity but low sensitivity [44]. These results are supported by additional studies that highlight the limited sensitivity of ICD-10 codes compared with approaches using objective clinical data for defining sepsis labels [45].

Niemantsverdriet *et al*. [46] argue that using ‘silver’ labels for model training may result in poorly trained models. In their study, ML models for sepsis diagnosis in the ED were trained with labels from 375 visits, determined by an 18-member Endpoint Adjudication Committee (EAC) comprising ED, internists and ICU specialists. The EAC accessed comprehensive EHR data, adhering to international guidelines (Sepsis-3) for sepsis identification. Two independent experts assessed all medical data, resolving discrepancies through a majority vote, with an additional expert consulted if needed. de Hond *et al*. [47] acknowledge the challenges of a labour-intensive EAC and expert biases in sepsis concepts. Nevertheless, an EAC, combining clinical experience, nuanced analysis of unstructured clinical data and adherence to recent guidelines, excels in capturing a comprehensive clinical picture. Training a model on EAC labels enhances diagnostic accuracy, reducing the risk of missing urgent sepsis cases requiring immediate attention. However, data quality remains suboptimal, with sepsis labelling acting as a bottleneck. In automated sepsis diagnosis in the ED, sensitivity is imperative to prevent fatal consequences from underdiagnosing. Flawed labels, unnoticed by algorithms, can significantly impact model accuracy, leading to inaccurate clinical diagnoses [47].

## Identifying sepsis cohorts

### Medical coding of sepsis

Physicians apply different criteria for sepsis to make a clinical diagnosis based on the sepsis definition used, while medical coders adhere to the ICD official guidelines for administrative coding. Variations in clinical diagnosis by physician speciality groups can influence administrative coding practices within an institution, potentially leading to variation in administrative data over time and between institutions. Accurate disease coding is crucial for facilitating reliable data for health service planning and research, as errors can result in significant inaccuracies [48].

The American Hospital Association Coding Clinic for the Fourth Quarter of 2017 mentions that a code should be assigned when the provider documents sepsis with associated acute organ dysfunction. However, this guideline may not always be explicitly evident in physician documentation practices [49]. Healthcare coders face challenges in administrative data applications due to evolving clinical definitions and unfamiliarity with specialized diagnostic results, such as pro-calcitonin levels [50]. Undercoding of sepsis, rooted in physician documentation practices, presents hurdles for accurate monitoring with ICD-10 codes. Coders may miss sepsis diagnoses when physicians use terms like ‘SIRS’ instead of ‘sepsis’, or when a narrow focus solely on the infection occurs [50]. Selective undercoding of milder sepsis forms may also happen when more clinically relevant or resource-intensive diagnoses take precedence [51].

Various ICD code strategies have been employed to identify sepsis cohorts from EHR data for retrospective analysis. However, there are limited data on the validity of different ICD coding methods for sepsis, and there is disagreement on the recommended approach [52]. Major strategies involve using ICD discharge diagnoses to identify sepsis hospitalization, as summarized in Table 1.

**Table 1.** Leveraging EHRs: major ICD coding strategies for identifying sepsis

Strategy	Description
Explicit	Sepsis-specific ICD codes, such as sepsis, severe sepsis, septic shock or septicaemia [79,82]
Angus (Implicit)	ICD-9-CM codes for infections and organ dysfunction within the same episode of care [83]
Dombrovskiy	Requires both ‘explicit’ sepsis codes and codes indicating organ dysfunction [84]
Martin	Uses specific ICD-9-CM codes for sepsis and a combination of diagnostic codes from ICD-9-CM and procedural codes from Current Procedural Terminology for organ failure [85]
Synchronous	Introduced in Australia, it involves coding synchronous diagnoses for infection and organ dysfunction, with a condition onset flag to exclude asynchronous infection and organ dysfunction cases [56,57]
Medicare ‘SEP-1’	Uses diagnostic ICD-9 codes, SIRS criteria and specific thresholds for organ dysfunction [86,88]

It is important to highlight that CMS’s General Equivalence Mapping files offer specific tables for both forward and backward mapping from ICD-9-CM to ICD-10-CM, noting that these mappings might not work in both directions [53].

Chan *et al*. [54] conducted a study on sepsis hospitalizations in U.S. academic medical centres, employing four classification systems: Angus, Martin, SEP-1 and Explicit. They found notable discrepancies in sepsis incidence, with Angus exhibiting the highest rates and Explicit the lowest. Most Explicit cases fell within the Martin and SEP-1 groups. Only a small proportion met the criteria of Martin or SEP-1 exclusively, and none met only the Explicit criteria. In their review, Liu *et al*. [55] found varied sensitivity and specificity of sepsis ICD-10 codes, indicating potential undercoding in administrative databases. On the accuracy front, Kumar *et al*. [52] identified the Martin criteria as the most reliable, although they acknowledged that study heterogeneity complicates interpretation. Additionally, synchronized coding emerged as a dependable approach in administrative datasets, emphasizing the need for comprehensive data integration for accurate epidemiological estimates [56,58].

### Objective clinical data of sepsis patients

Prior to 2017, estimates of the sepsis burden in the USA relied on administrative codes, indicating an increasing incidence and decreasing mortality rates. However, during this period, there was a noticeable rise in sepsis coding, while coding for typical underlying infections remained consistent or decreased. This suggests that coding practices might have been influenced by heightened awareness of sepsis and financial incentives, potentially rendering them unreliable for surveillance [59]. The ongoing controversy surrounding ICD-10 codes for sepsis monitoring involves changes in the definition of sepsis, evolving coding practices, and a wide array of potentially suitable codes. These challenges emphasize the need for further investigation and standardization [60,62].

In 2017, a consortium funded by the Centres for Disease Control and Prevention (CDC) introduced two innovative sepsis definitions specifically tailored for surveillance purposes. These definitions utilized objective clinical data elements extracted from EHRs to enhance accuracy and reliability.

**Fig. 3.**
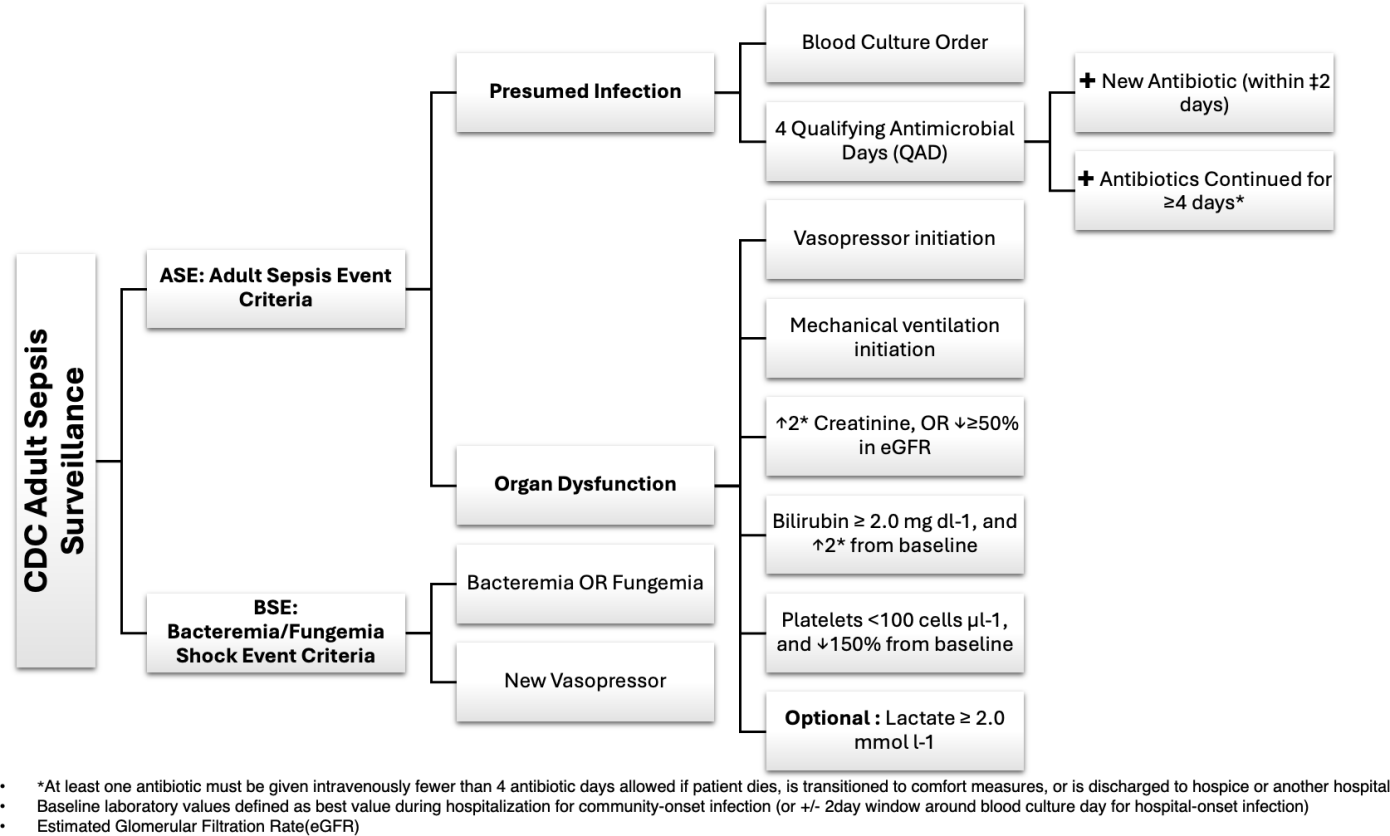
CDC ASE definition [59].

The first definition, the CDC’s Adult Sepsis Event (ASE), is based on the Sepsis-3 framework and involves two main components: presumed infection and organ dysfunction. Presumed infection requires the presence of both a blood culture order and four qualifying antimicrobial days. Organ dysfunction includes at least one of the following criteria: vasopressor initiation, mechanical ventilation, increased creatinine or decreased estimated glomerular filtration rate, elevated bilirubin, decreased platelets and optionally elevated lactate levels. Surveillance of ASE necessitates access to microbiology, medication administration, laboratory and administrative (ICD) coding data [63]. Fig. 3 illustrates the CDC’s Adult Sepsis Surveillance criteria, including the ASE elements.

The CDC’s ASE surveillance definition is based on the Sepsis-3 framework, involving suspected infection coupled with organ dysfunction. Surveillance of ASE necessitates access to microbiology, medication administration, laboratory and administrative (ICD) coding data (Fig. 3) [63]. In cases where procedure codes are unavailable, clinical records of mechanical ventilation serve as an alternative option. The second definition introduced by the CDC, the Bacteremia/Fungemia Shock Event (BSE), offers a simplified approach targeting a narrower patient group with higher mortality rates. It relies on positive blood cultures (excluding common contaminants) and the use of vasopressors to identify clinically significant infections and hypotension. BSE utilizes minimal data components, focusing on microbiology and medication administration records [59]. Fig. 3 also illustrates the BSE criteria, which include positive blood cultures and new vasopressor initiation.

Rhee *et al*. [24] validated the CDC’s ASE definition in 6% of hospital admissions, revealing 69.7% sensitivity, 98.1% specificity, 70.4% positive predictive value and 98.0% negative predictive value using the Sepsis-3 criteria as the reference standard. In another study, Rhee *et al*. compared how sepsis patients were identified using the CDC’s ASE (referred to as ‘eSOFA’) versus the more complex SOFA score, utilizing EHR data from various hospitals. The eSOFA approach identified a smaller yet more severely ill sepsis patient cohort compared with the SOFA score method. These findings support considering ASE as a practical and consistent tool for automated sepsis surveillance by both hospitals and public health agencies [64]. In a subsequent unpublished analysis, Rhee *et al*. identified BSE in 0.48% of hospital admissions (compared with 6.0% with ASE). Compared with 15% of ASE, 35.4% of BSE events resulted in death during hospitalization. It demonstrated 6.0% sensitivity and 100% positive predictive value using the Sepsis-3 criteria [59].

### Machine learning and EHRs

Traditionally, phenotypes were determined using rule-based algorithms that included inclusion and exclusion criteria developed by clinical and informatics professionals. These rule-based approaches are effective for phenotypes characterized by distinct diagnosis and procedure codes. In contrast, ML methods, unlike rule-based approaches, automate and generalize the integration of various information from patient records to enhance phenotypic characterization in a more automated and generalizable manner [65].

ML and cloud computing hold the potential to significantly improve the detection and monitoring of emerging diseases using secondary health-related data [66]. Additionally, research conducted through these applications can provide new insights into the origins and effects of diseases [67]. The phenotyping process typically involves four steps: (1) preparing the data, (2) developing the algorithm, (3) evaluating the algorithm and (4) applying the algorithm [68].

### Machine learning methods and sepsis

#### Supervised learning

Supervised learning involves algorithms trained with labelled data to define phenotypes. Common techniques include random forest (RF), logistic regression (LR) and support vector machines. These methods are suitable for predictive tasks but face scalability challenges due to the time and resources required for accurate labelled data acquisition [65,69].

Goh *et al*. [70] developed the Sepsis Early Risk Assessment algorithm, an artificial intelligence model for diagnosing and predicting sepsis using both structured data and unstructured clinical notes. They employed latent Dirichlet allocation (LDA) to convert unstructured text into a numerical format. The diagnostic algorithm uses a voting ensemble ML approach, with additional comparisons using bagging and gradient-boosted trees as alternative classifiers. If a patient is classified as not having sepsis, the early prediction algorithm assesses the risk of sepsis in the next 4, 6, 12, 24 and 48 h using the voting ensemble ML method.

Niemantsverdriet *et al*. [46] emphasize that ML should not only be used for developing diagnostic tools. They employed LR, L1 regularization and RF to identify diagnostic predictors and their importance in sepsis. The evaluation included 95 variables from demographic/vital, laboratory and advanced haematological groups, involving univariate testing and training ML models.

#### Semi-supervised learning

Semi-supervised learning reduces the need for extensive labelled data by training on both labelled and unlabelled data. This approach is particularly useful for phenotyping when labelled data are scarce. It involves three main methods: the first, using ‘silver-standard labels’ extracted from patient records to select features; the second, generating pseudolabels from a small set of labelled data to train a supervised model on a larger unlabelled dataset; and the third, directly integrating unlabelled data into the algorithm by modifying the loss function [68,71].

Bashiri *et al.* [72] highlight the importance of early infection identification for better outcomes. Model development typically relies on manual chart reviews, limiting sample size. Their multicentre study compared semi-supervised and transfer learning algorithms to manual chart review for infection identification across six hospitals, using ‘gold-standard’ labels from chart review and ‘silver-standard’ labels from non-chart-reviewed patients based on the Sepsis-3 criteria. The study aimed to identify methods with optimal discrimination and calibration, evaluating variables like demographics, unit type, vital signs, interventions and laboratory results. In nearly 3,000 chart-reviewed patients, semi-supervised and transfer learning models showed similar discrimination to baseline XGBoost, with improved calibration in transfer learning.

Halpern *et al*. [73] introduced ‘Learning with Anchors’ a semi-supervised method for developing a phenotype library with 42 publicly available EMR-based definitions. The method employs both structured and unstructured data, using ‘anchor’ observations that fulfil two criteria for phenotype prediction: high positive predictive value and conditional independence, identified through domain expertise. Each phenotype is defined by its anchors, which can be specified as ICD-9 codes, medications (history or dispensed) or free text. For septic shock, medication dispensing records proved more informative than free text, unlike other acute phenotypes such as infection, pneumonia and cardiac causes. Notably, pneumonia and septic shock achieved a high area under the curve values (*>*0.95), indicating easier detection compared with other conditions.

#### Weakly-supervised learning

In phenotyping, there is a growing trend towards weakly-supervised learning, which aims to reduce reliance on gold-standard labelled data. This approach uses ‘silver-standard’ labels, which are chosen by domain experts as highly predictive yet imperfect proxies for the gold-standard and can be easily extracted from all records [74]. An example of a silver-standard label in sepsis phenotyping could be specific diagnosis codes, such as ICD-10 codes for sepsis.

Two main approaches in weakly-supervised learning include one that assumes a mixture model for the silver standard label, representing phenotype cases and controls, and another that directly trains supervised models using silver standards [68]. However, most weakly-supervised methods encounter the challenge of selecting an optimal cutoff value for generating the best classifier and may falter for episodic phenotypes inconsistent in ICD and natural language processing data. Addressing these limitations, Nogues *et al*. [75] introduced a label-efficient, weakly semi-supervised deep learning algorithm for EHR phenotyping. This method classifies patient-level disease status through stages involving generating silver-standard labels, deriving enhanced silver-standard labels using weakly supervised deep learning, and obtaining final predictions and classifiers through supervised learning with minimal gold-standard labels.

#### Unsupervised learning

Unsupervised learning identifies meaningful patterns, such as disease progression clusters or subtypes, from unlabelled data. This method is essential for discovering new phenotypes and identifying sub-phenotypes, co-occurring conditions and disease progression patterns. Common techniques include LDA, *K*-means and the unweighted pair group method with arithmetic mean hierarchical clustering [68]. Disease subtyping, an idea originating from Hippocrates, enables the recognition of subgroups among individuals with a shared disease, characterized by common traits. Accurate phenotyping is crucial for precision medicine, and there is a growing shift from clinically driven disease descriptions to letting health data convey insights.

Li *et al*. [76] conducted a scoping review of 17 clinical studies on sepsis or septic shock, employing various clustering methods for phenotyping and identifying clusters that predict clinical outcomes. The review found that while all studies identified clusters predicting clinical outcomes like mortality, only seven studies identified clusters predicting treatment response. Seymour *et al.* [77] used consensus *K*-means clustering to categorize sepsis into four phenotypes (*α*, *β*, *γ* and *δ*), each with unique demographic features, varied biochemical presentations, correlated host-response patterns and diverse clinical outcomes.

Techniques like principal component analysis (PCA) and t-distributed Stochastic Neighbour Embedding (t-SNE) enhance data interpretation. PCA reduces dimensionality for clearer visualisation, while t-SNE handles non-separable data, ideal for 2D and 3D visualization.

Hu *et al*. [78] aimed to identify and compare subphenotypes in a large sepsis cohort. They distinguished two subphenotypes, A and B, using 11 clinical variables assessed within 24 h of ICU admission. Subphenotype B was characterized by higher levels of lactate, glucose, creatinine, white blood cell count, sodium, and heart rate, and lower body temperature, platelet count, systolic blood pressure, haemoglobin and PaO2/FiO2 ratio. Notably, in-hospital mortality was significantly higher in subphenotype B than in subphenotype A. The researchers employed PCA for visualization, creating two-dimensional images to highlight differences before and after clustering.

## Conclusion

This review provides a comprehensive overview of health data-driven research and ML-based phenotyping for sepsis, summarizing the current landscape and examining the associated challenges. Sepsis, as a syndrome, presents distinct challenges due to varying clinical opinions, multiple definitions and its high dependence on individual host responses, making it unpredictable.

Clinicians often use diverse approaches, resulting in varied reflections on the data. Amidst these complexities, the critical role of clinical coders is often overlooked. Coders are tasked with making decisions and assigning ICD codes to patients on discharge based on an often unclear clinical picture. Assigning a code for sepsis can be as challenging as identifying it. Additionally, the primary application of ICD-coded data for funding and reimbursement purposes has contributed to limitations in the reliability of coded data for research.

Electronic health data present various challenges due to their dynamic and disparate nature, large availability, incompleteness, privacy concerns, limited access and labelling issues. Researchers use ICD codes, laboratory values, clinical notes and medications to identify sepsis cohorts, aiming to improve sensitivity and specificity.

For short-lived syndromes like sepsis, the optimal solution involves modifying the electronic medical applications used by institutions. Introducing a clear flag that reflects physician decisions can provide high-quality labelled data for researchers. This approach supports in-depth analyses, allowing a better understanding of sepsis biomarkers and causal inferences. Moreover, this strategy improves the reliability of data for further analyses, enabling evaluations of different sites in terms of decision-making accuracy, effectiveness and adherence to sepsis pathways, definitions and survival campaigns. Additionally, a nationwide sepsis registry would be beneficial, offering a valuable resource for researchers to explore sepsis from various perspectives.

## Abbreviations

ASE: adult sepsis event
BSE: bacteremia/fungemia shock event
BSIs: bloodstream infections
CDC: centres for disease control and prevention
CMS: centers for medicare and medicaid services
EAC: endpoint adjudication committee
EHR: electronic health record
EMR: electronic medical record
ESICM: European Society of Intensive Care Medicine
ICD: International Classification of Diseases
ICU: intensive care unit
LDA: latent Dirichlet allocation
LR: logistic regression
ML: machine learning
PCA: principal component analysis
RF: random forest
RTW: return-to-work
SCCM: society of critical care medicine
SIRS: systemic inflammatory response syndrome
SOFA: sequential organ failure assessment
t-SNE: t-distributed Stochastic Neighbour Embedding
WHO: World Health Organization
